# What can Written-Words Tell us About Lexical Retrieval in Speech Production?

**DOI:** 10.3389/fpsyg.2015.01982

**Published:** 2016-01-06

**Authors:** Eduardo Navarrete, Bradford Z. Mahon, Anna Lorenzoni, Francesca Peressotti

**Affiliations:** ^1^Dipartimento di Psicologia dello Sviluppo e della Socializzazione, Università di PadovaPadova, Italy; ^2^Department of Brain and Cognitive Sciences, University of RochesterRochester, NY, USA; ^3^Department of Neurosurgery, University of Rochester Medical CenterRochester, NY, USA; ^4^Center for Language Sciences, University of RochesterRochester, NY, USA

**Keywords:** speech production, word reading, semantic context effect, lexical access, picture naming

## Abstract

In recent decades, researchers have exploited semantic context effects in picture naming tasks in order to investigate the mechanisms involved in the retrieval of words from the mental lexicon. In the blocked naming paradigm, participants name target pictures that are either blocked or not blocked by semantic category. In the continuous naming task, participants name a sequence of target pictures that are drawn from multiple semantic categories. Semantic context effects in both tasks are a highly reliable phenomenon. The empirical evidence is, however, sparse and inconsistent when the target stimuli are printed-words instead of pictures. In the first part of the present study we review the empirical evidence regarding semantic context effects with written-word stimuli in the blocked and continuous naming tasks. In the second part, we empirically test whether semantic context effects are transferred from picture naming trials to word reading trials, and from word reading trials to picture naming trials. The results indicate a transfer of semantic context effects from picture naming to subsequently read within-category words. There is no transfer of semantic effects from target words that were read to subsequently named within-category pictures. These results replicate previous findings (Navarrete et al., 2010) and are contrary to predictions from a recent theoretical analysis by Belke (2013). The empirical evidence reported in the literature together with the present results, are discussed in relation to current accounts of semantic context effects in speech production.

## Introduction

One of the most widely investigated issues by researchers interested in language production concerns the nature of the processes involved in the retrieval of words from the speaker’s memory system. Researchers agree on two general architectural parameters about lexical access. The first universally agreed-upon parameter is that what determines word retrieval is the level of activation of the corresponding lexical representation, in the sense that the word that is ultimately produced was the most highly activated lexical representation at the moment that it was retrieved for production. The second assumption shared by models of speech production is that activation spreads from the semantic system to the lexical system, and a single concept cannot be activated alone, but rather spreads activity to a cohort of related concepts. The implication is that not only the target word is activated, but also a cohort of semantically related words; thus, the target word must be retrieved against a backdrop of activated but non-target words (e.g., Dell, 1986; Caramazza, 1997; Levelt et al., 1999; Rapp and Goldrick, 2000).

Based on these assumptions, a straightforward empirical approach for exploring lexical retrieval consists in manipulating the semantic context within which speakers retrieve words in naming tasks. Many empirical studies have adopted this approach and there is compelling evidence that speaking is modulated by semantic context: the time and the accuracy of word production is affected by the semantic relationship between the targets, the to-be uttered words, and non-target and potentially task irrelevant stimuli. Broadly speaking two types of semantic context manipulations can be distinguished, one in which semantic context is manipulated at the *intra-trial level* and one in which semantic context is manipulated at the *inter-trial level.* For *intra-trial* semantic context manipulations, participants have to name a target stimulus while ignoring the presentation of a distractor element that can be semantically related or unrelated with the target. The distractor element appears within the same trial, that is, simultaneously with, or slightly before or after, the target itself. This is the type of manipulation behind Stroop-like interference paradigms, such as the picture-word interference (e.g., Rosinski, 1977; Lupker, 1979; La Heij, 1988; Schriefers et al., 1990; Damian and Bowers, 2003; Roelofs, 2003; Finkbeiner and Caramazza, 2006) and the word–word interference tasks (Glaser and Glaser, 1989; La Heij et al., 1990; Roelofs et al., 2013; Treccani and Mulatti, 2015).

One of the most widely exploited experimental approaches for investigating lexical retrieval has been done in the tradition of *intra-trial* manipulations, and in particular, the picture-word interference task. In this task, participants are required to name pictures while ignoring the presentation of a distractor word (for reviews see Mahon et al., 2007; Navarrete and Mahon, 2013; Spalek et al., 2013). One concern with that paradigm is that in order to understand how target words are retrieved, bridging assumptions are required about how distractor stimuli are processed in the system. To date, there is still no consensus on this issue, as debate continues regarding (i) how distractors are excluded from production, (ii) and whether, and if so how, distractor exclusion affects retrieval of the target words (e.g., Damian and Bowers, 2003; Dhooge and Hartsuiker, 2010; Roelofs et al., 2011; Mahon et al., 2012; Mulatti and Coltheart, 2012, 2014; Finocchiaro and Navarrete, 2013; Hantsch and Mädebach, 2013; Hutson et al., 2013; Mädebach and Hantsch, 2013; Navarrete and Mahon, 2013; Roelofs and Piai, 2013; Starreveld et al., 2013; Mahon and Navarrete, 2014). More recently, researchers have focused on *inter-trial* semantic manipulations.

In *inter-trial* manipulations, the goal is to explore how lexical retrieval (i.e., on trial *n*) is modulated by having retrieved semantically related or unrelated words on preceding trials (e.g., on trials *n-1, n-2, n – 3…*). It has been shown that picture naming requires more time (and is more prone to errors) when semantic coordinate words have been retrieved some trials before. For instance, participants name pictures (e.g., shark) more slowly when some trials before a semantic coordinate word (e.g., whale) is named as a response to a written definition, compared to when a non-semantic coordinate was previously named (e.g., volcano) (Wheeldon and Monsell, 1994). Semantic costs are also reported when, instead of naming written definitions, participants name pictures (e.g., Brown, 1981; Howard et al., 2006). In contrast, when semantic coordinate pictures are presented on two *consecutive* trials, semantic facilitation instead of a semantic cost (i.e., interference) is observed. That is, a picture on trial *n* is named faster when a semantic coordinate picture is named on trial *n-1* compared to when a semantically unrelated picture was named on trial *n -1* (e.g., Huttenlocher and Kubicek, 1983; Lupker, 1988; Biggs and Marmurek, 1990). While semantic interference is a long lasting phenomenon that persists over several trials, semantic facilitation is a short lasting phenomenon that is observed only for consecutive trials (e.g., Damian and Als, 2005; Navarrete et al., 2012, 2014).

Aside from the distinction between *intra-trial* and *inter-trial* semantic context manipulations, another critical dimension concerns the format of the target stimuli, which can be either pictures or printed words. The distinction between picture and word format is of critical relevance because while naming a target picture is a semantically mediated task, in the sense that the lexicon is accessed through visual and semantic processing, word reading can be achieved independently of semantic mediation and be resolved through direct links between orthographic and phonological representations (i.e., via sub-lexical processing; see for instance Coltheart, 2004). This does not mean that there is no semantic activation in reading and that some amount of activation will be propagated through to the semantic system (Coltheart et al., 1993, 2001; Perry et al., 2007); rather, word naming can be accomplished without semantic mediation. Perhaps the neuropsychological pattern of word-meaning blindness is the most intuitive evidence of this. Patient JO reported by Lambon Ralph et al. (1996), was able to read aloud words and non-words and was perfect on visual lexical decision tasks. Access to meaning was also normal for spoken words and from objects. Interestingly, JO was severely impaired at comprehending written words during silent reading. Further evidence that printed word stimuli can bypass semantic mediation comes from word translation tasks in bilingual speakers (Kroll and Stewart, 1994; Navarrete et al., 2015).

The advantage of *inter-trial* manipulations is that, as described above, there is no distractor element that needs to be excluded from production. In this case, the semantic context manipulation must be understood in terms of temporal extension in the activation dynamics of the lexical system, i.e., as in a form of memory. Elsewhere we have reviewed the principal phenomena observed with *inter-trial* manipulations in picture naming tasks, that is, semantic facilitation and semantic interference effects with target pictures (Navarrete et al., 2012, 2014). In the current review, we focus on experimental results with target words and compare them with those obtained with target pictures. As we will see, empirical evidence from experiments in which the target stimuli are printed words is sparse and inconsistent. In the last section of this paper, we aim to empirically resolve, at least in part, this inconsistency by replicating the results of a previous study (Experiment 3, Navarrete et al., 2010). Before, and in order to better situate the implications of the reviewed empirical findings, in the next section we briefly review the main theoretical accounts of long lasting *inter-trial* semantic interference.

### Theoretical Accounts of Long Lasting Inter-Trial Semantic Interference

Two main approaches have been proposed to account for long lasting *inter-trial* semantic interference. Oppenheim et al. (2007, 2010) have implemented an incremental learning mechanism by which semantic-to-lexical connection weights are adjusted after each naming event (see also, see Damian and Als, 2005). The production of a word as a response to a target picture strengthens the connections between the semantic and lexical representations of that word (e.g., *cat*) and, at the same time, weakens the connections between semantic and lexical representations of semantic coordinates of that word (e.g., *dog*, *horse*). When on a subsequent trial a semantic coordinate item has to be retrieved (e.g., *dog*), naming latencies will be longer because of the weakened semantic-to-lexical connections (see also Vitkovitch and Humphreys, 1991; Navarrete et al., 2010, 2012, 2014; Kleinman et al., 2015). The second approach is based on the hypothesis that lexical retrieval (i.e., selection) is a competitive process, so that the time required to retrieve a word depends on the levels of activation of other activated but non-target words (e.g., Roelofs, 1992, 1993; Levelt et al., 1999). When a word is produced as a response to a picture stimulus, it retains lexical activation for a certain period of time, making it a stronger competitor when, on subsequent trials, a semantic coordinate has to be retrieved. For instance, according to Howard et al. (2006), long lasting *inter-trial* semantic interference arises due to the convergence of three properties: priming, shared activation and competitive lexical selection. In their model, Howard et al. (2006) implement competition by lateral inhibition between lexical candidates, that is, each lexical unit (i.e., lemma in their model) inhibits other lexical units in proportion to its own activation level.

Within the competition account, some researchers argue that semantic interference should emerge in all circumstances which require the retrieval of a lexical representation (e.g., Damian et al., 2001; Vigliocco et al., 2002); other researchers argue that semantic interference emerges only when lexical selection is conceptually mediated (e.g., Howard et al., 2006; Belke, 2013). For instance, somewhat different from Oppenheim and colleagues’ view, Belke (2013) implemented the incremental learning mechanism at the conceptual level in the links between semantic features and lexical semantic representations. In that framework, semantic interference originates at the conceptual level, although its locus remains at the lexical level by a mechanism of selection by competition.

### Long Lasting Inter-Trial Semantic Interference with Target Words: Empirical Findings and Theoretical Implications

There exists compelling evidence that long lasting *inter-trial* semantic interference emerges when lexical retrieval is semantically mediated, as for instance in picture naming or in definition naming tasks (Wheeldon and Monsell, 1994; for review see, Navarrete et al., 2014). By contrast, studies exploring semantic effects using printed word stimuli as targets show a more complex pattern. In their influential study, Damian et al. (2001) explored semantic interference in the blocked naming paradigm. In this task, first introduced by Kroll and Stewart (1994), participants are slower to name pictures if they were grouped into a block of all within-category items (e.g., *cat, dog, horse*) compared to blocks of items from different categories (e.g., *cat, table, lemon*). Damian et al. (2001) introduced several important changes to the original blocked naming task devised by Kroll and Stewart (1994). Of particular relevance, target words were presented instead of target pictures and participants were asked to name them either accompanied by the corresponding grammatical gender-marked determiner or in a standard reading task. Grammatical gender is a syntactic feature of nouns and cannot be predicted from conceptual properties (e.g., Navarrete and Costa, 2009), except when it correlates with conceptual properties as in the case of natural gender (e.g., Vigliocco and Franck, 1999). Therefore, even though a printed word is presented, the lexical-syntactic representation corresponding to the word must be retrieved in order to retrieve its gender. Of critical relevance, the modification introduced by Damian et al. (2001) allows for the testing of different accounts regarding semantic interference effects in speech production. The logic underlying the use of this task is that if semantic interference is ascribed to lexical processes, the effect should be present in a determiner + word naming task. In contrast, it may be argued that if semantic interference emerges because of adjustments to the mappings from semantic representations to a specific lexical representation (i.e., the target word), and such adjustments are not required when the target is a word, word stimuli should not elicit semantic interference, even in the case of a determiner + word naming task (for extended discussion of these issues, see Navarrete et al., 2010). Furthermore, it may also be argued that semantic interference emerges through lexical selection by competition only when the task at hand implies conceptual mediation; therefore, if determiner + word naming does not mandatorily require conceptual retrieval, word stimuli should not elicit semantic interference (Howard et al., 2006; Belke, 2013).

Damian et al. (2001) observed interference with picture stimuli, replicating Kroll and Stewart (1994). Critically, semantic interference was also observed with printed words in the determiner + word naming task, but not in a bare noun naming task. Indeed, when participants read the words without the determiner, a semantic facilitation effect emerged: response times were faster in the related blocks than in unrelated blocks. Damian et al. (2001) interpreted this pattern as congruent with the explanation of semantic interference in terms of competition during the selection of the syntactically specified word representation (i.e., lemma selection, in their model; for similar arguments see also Vigliocco et al., 2002; Roelofs, 2006). Because grammatical gender is a syntactically specified lexical feature, lemma selection is required to perform the task and interference would emerge as a consequence of increased competition for lemma selection in blocks containing within-category word stimuli, compared to blocks containing word stimuli from different categories. In contrast, competition would not arise in a bare noun naming task because speakers can read target words by accessing word form representations via a route that bypasses semantic and lemma representations (Damian et al., 2001). In sum, the two printed word manipulations introduced by Damian et al. (2001) produce opposing semantic effects: semantic facilitation in bare noun production and semantic interference in determiner + word naming production. Below, we focus on these two effects.

Somewhat in contrast to Damian et al. (2001) conclusion, other studies have reported semantic interference induced by printed words even when syntactic information is not required to perform the task at hand, as in bare noun naming. For instance, participants in the study by Vitkovitch et al. (2010) first read a sequence of words and then, shortly afterwards, they named a sequence of pictures. The results showed a semantic interference effect on picture naming, such that picture naming times were longer if some trials before semantic coordinate words had been read, compared to when unrelated words had previously been read (see also Tree and Hirsh, 2003; Vitkovitch et al., 2006; Vitkovitch and Cooper-Pye, 2012). Vitkovitch et al. (2006) interpreted this word-to-picture semantic interference as congruent with competition during name retrieval, so that the relative activation of competitors (i.e., the word stimuli) slows down the selection of the picture name. Critically, as this was a bare noun-reading task, no syntactic information had to be retrieved in order to perform the task; thus, according to the framework of Damian et al. (2001), no semantic interference should be expected. In other words, there should be no transfer of semantic interference from word reading to picture naming. Furthermore, and in contrast with the facilitation effect reported by Damian et al. (2001), Janssen et al. (2011) have reported a small, but reliable, semantic interference effect in a blocked naming task with bare-noun reading.

On the other hand, the semantic interference effect observed by Damian et al.’s (2001) study in the determiner + word naming can be contrasted with the findings of Navarrete et al. (2010) and Belke (2013). Belke (2013) did not observe semantic interference in the determiner + word naming task using the same language, procedure and task as was used by Damian et al. (2001). Navarrete et al. (2010) also failed to observe semantic interference using target words in the continuous naming paradigm, a paradigm similar to the one used by Vitkovitch et al. (2010). In the continuous naming task, participants are presented with a sequence of items (pictures or words) from diverse semantic categories in a (seemingly) random order. A reliable phenomenon is the cumulative semantic cost: picture naming times increase for every successive within-category item that is named. That is, the naming latency for each item is determined by the total number of items from the same category that have been already named (for early work see Brown, 1981; for more recent work see Howard et al., 2006; Costa et al., 2009; Alario and Martin, 2010; Runnqvist et al., 2012; Belke and Stielow, 2013; Schnur, 2014; Navarrete et al., 2015). Navarrete et al. (2010) observed the cumulative semantic cost using pictures as targets, but no cumulative cost in a determiner + word naming task. In a further experiment, participants were presented with a sequence of intermingled words and pictures and named them (all) along with the corresponding gender-marked determiner. In that experiment, a semantic interference effect was obtained for both words and pictures, but only when the preceding within-category items were pictures, and not when the preceding within-category items were words (but see Belke, 2013, and below). Navarrete et al. (2010) concluded that naming a picture entails adjustments to semantic-to-lexical connections, specifically, incremental weakening of the semantic-to-lexical connections for semantic coordinates of the target word. Such adjustments affect the time required to access lexical representations on subsequent within-category trials, irrespective of their format (i.e., picture or word). In contrast, Navarrete et al. (2010) argued that, naming a word does not entail incremental weakening adjustments to semantic-to-lexical mappings, and therefore, the time required to access lexical representations on subsequent within-category trials is unaffected by semantic context (again, irrespective of its format, i.e., picture or word).

### Interim Summary

As outlined above, semantic context effects in picture and word naming experiments is a common and straightforward approach to explore lexical retrieval during speech production. While empirical evidence regarding picture naming research is relatively congruent (see for instance, Navarrete et al., 2014), this is not so within word naming research. It is evident from the previous paragraphs that there is no simple answer as to whether word stimuli are able to elicit long lasting *inter-trial* semantic interference in language production. Certainly, differences between blocked naming and continuous naming designs may be relevant for explaining divergent findings. However, the lack of replication within the same paradigm remains problematic. For instance, in contrast with what was observed by Damian et al. (2001) in the semantic blocked naming paradigm, Belke (2013) did not report semantic interference in the determiner + word naming task. In addition, Janssen et al. (2011) using the same paradigm, reported semantic interference in bare noun production. Further experimental evidence is therefore needed in order to pinpoint which are the relevant factors in determining whether semantic interference with word targets is observed. Here we seek to provide some of this evidence by focusing on the contrasting experimental results within the continuous naming task.

Recently, Belke (2013) failed to replicate the transfer of semantic interference from pictures to words that we reported in a previous determiner + word/picture naming study (Navarrete et al., 2010; see above). In our original experimental design, for each semantic category, four items were presented within the same format (e.g., picture format) and one in a different format (in this case, word format). The deviant condition referred to those items presented in a different format than the other four within-category items, while the non-deviant condition referred to those items presented in the same format as the other four within-category items. The results of our Experiment 3 indicated semantic interference for both pictures and words when the preceding within-category items were pictures, and no semantic interference effect when the preceding within-category items were words. Belke (2013) argued that those results may have been due to uncontrolled switching costs. As picture and word stimuli were presented randomly intermingled within the naming sequence, there were, within the sequence, switch and non-switch trials, as a function of whether the previous trial contained a same-format stimulus or a different-format stimulus, respectively. Belke did not explain how the switch cost might account for our results; nevertheless it might be surmised that semantic interference could be ‘confused’ with switch costs. In order to control for such possible confusion, here we first reanalyze our data (Navarrete et al., 2010; Experiment 3), distinguishing switch from non-switch trials.

Half of the trials were switch trials and the other half were non-switch trials. At the same time, half of the pictures were presented on switch trials and half on non-switch trials. The same was the case for word stimuli. Mean latencies, split by Switch Type, are reported in **Table 1**. As can be seen, transfer effects indeed were modulated according to whether or not the trial was a switch trial—but importantly, the critical finding is present for non-switch trials and absent on switch trials. Deviant determiner + word naming trials (i.e., word naming that followed within-category picture naming trials) were slower than non-deviant determiner + word naming trials (i.e., word naming that followed within-category word naming trials), but only when the trials were non-switch trials [22 ms, *t*(19) = 3.98, *p* < 0.002]. By contrast, such an effect was absent for switch trials (3 ms, *t* < 1), indicating that switching the format of target presentation canceled out (or decreased) the transfer of semantic interference from pictures to words. Switching was also a relevant factor in the determiner + picture naming condition. In our original study, the lack of semantic interference transfer from within-category words to pictures was defined as the difference between deviant and non-deviant determiner + picture naming trials. Specifically, if there is no transfer from determiner + word naming to determiner + picture naming trials, deviant determiner + picture naming trials (those that followed within-category word trials) should be named faster than non-deviant determiner + picture naming trials (those that followed within-category picture trials). This prediction was confirmed in the Navarrete et al. (2010) study. The re-analysis performed here shows that switching was again a critical variable, such that the difference between deviant and non-deviant determiner + picture naming trials was reliable for non-switch trials only [54 ms, *t*(19) = -4.93, *p* < 0.001]. No differences between deviant and non-deviant determiner + picture naming trials were observed for switch trials (2 ms, *t* < 1).

**Table 1:** Mean naming averages (RT in ms), standard errors (SE, in ms) for Non-deviant and Deviant trials in the determiner + picture naming and determiner + word naming trials, broken down by trial type (i.e., Non-switch and switch), of the Experiment 3 of Navarrete et al. (2010).

Trial type	Non-deviant		Deviant		
	*RT*	*SE*	*RT*	*SE*	
Determiner + picture naming					*Transfer Effect*
Non-switch trials	747	18	693	18	*54*
Switch trials	714	19	712	17	*2*
*Switch effect*	*33*		-*19*		
Determiner + word naming					
Non-switch trials	513	13	535	14	-*22*
Switch trials	519	13	516	16	*3*
*Switch effect*	-*6*		*19*		

*The Transfer Effect is calculated as the difference between Non-deviant minus Deviant per each of the 4 sub-conditions (see Navarrete et al., 2010, per details).*

In sum, the re-analysis suggests that switching the format of the target may indeed affect the transfer/absence of semantic interference in the continuous naming task. However, contrary to the hypothesis of a confound between switch costs and semantic interference, semantic interference was obtained only for non-switch trials, leaving intact the conclusion reached in our previous study, and undermining the concerns raised by Belke (2013). However, and nonetheless, further empirical work is called for in order to understand how switching between formats could modulate semantic interference in the continuous naming tasks. Altogether, our re-analysis contrasts with the results reported by Belke (2013), who did not find transfer of semantic interference effect in any direction. In the present study, we aim to replicate the interaction between the transfer of semantic interference and the target format (picture or word) in the continuous naming task, while attending to any effects of switch costs.

Testing the transfer of semantic interference has relevant implications for models of lexical retrieval. Under the assumption that semantic interference effects originate from competition at the lexical level (e.g., Damian et al., 2001; Vigliocco et al., 2002; Vitkovitch et al., 2010; Vitkovitch and Cooper-Pye, 2012), there should be transfer of semantic interference in both directions, that is, independently of stimuli format (i.e., pictures or words). In contrast, according to the competitive account of Belke (2013), no transfer of semantic interference effects are expected because determiner + word naming trials do not involve conceptual processing. Finally, according to Navarrete et al. (2010), there should be transfer of semantic interference from picture to word naming trials but not from word to picture naming trials.^1^

## The Present Study

The goal of this experiment was to replicate the interaction between stimulus format and the transfer of semantic cost in the continuous naming task, originally reported by Navarrete et al. (2010, Experiment 3). Four conditions were included. In two conditions four within-category items were presented in the same format: in a picture format in the PPP-P condition, and in a word format in the WWW-W condition^2^. In the other two conditions, the item located in the fourth within-category position was presented with a different format than the previous three within-category items: In the PPP-W condition the first three within-category items were presented in a picture format and the fourth item in a word format, and in the WWW-P condition the first three within-category items were presented in a word format and the fourth item in a picture format. In order to avoid potential effects of switching, care was taken that all critical trials were in the same format as the preceding trial of the sequence. In other words, all critical trials were non-switch trials, such that issues having to do with switch costs simply do not arise.

### Method

#### Participants

Forty native Italian speakers (undergraduate students at the University of Padova) gave written informed consent to take part in the experiment. Ethical approval was granted by the Ethical Committee for the Psychological Research of the University of Padova (protocol number: 1361, title: *Mechanisms of Word Retrieval in Spoken Language Production*).

#### Materials

One hundred and forty-eight items were selected. Items were presented in black upper case letters (Times Roman, Regular, 24 point) and as color photographs taken from the Internet and sized to fit within a square of 400 × 400 pixels, for the word and picture format respectively. Eighty of the items belonged to 20 different semantic categories, with four items from each category. The remaining items were fillers that did not belong to the same categories as the experimental items.

#### Design

The 148 items were randomly inserted into a sequence with the following constraints. Items from each category were separated by lags of 2, 4, or 6 intervening items. Each lag was used the same number of times in the sequence (i.e., 20). The first five items of the sequence were filler items. Filler items and the order of the categories in the sequence were randomly assigned with the following constraints. Five categories were assigned to each of the four experimental conditions (i.e., PPP-P, PPP-W, WWW-W, WWW-P). Half of the filler items were presented in picture format and the other half in word format. Thus, the sequence contained 74 trials in picture format and 74 trials in word format. There were a total of forty-eight switch trials (i.e., formats in trial *n* and *n-1* were different) and ninety-nine non-switch trials (i.e., same format on trials *n* and *n-1*). Switch trials always entailed filler items: the preceding trial of a critical trial was always presented in the same format as the critical trial.

Once the first sequence was created, the same structure was used to generate nineteen new sequences. In generating these first 20 experimental sequences, it was ensured that each specific category occupied a different position across the 20 sequences. The critical items within each category were presented equally at each of the four ordinal positions (i.e., across this group of 20 sequences each critical item was presented a total of five times in each within-category ordinal position, 1 to 4). Finally, a new group of 20 sequences was created from this first group of 20 sequences by changing the format of presentations of all the items (so that each sequence had a paired sequence with the same item presentation but varying only the format of presentation of the items). There were a total of 40 experimental sequences. Each participant was presented with two sequences, one sequence of the first group and one sequence of the second group. Care was taken that the same participant was not presented with paired sequences. Each of the 40 experimental sequences was used twice across all the participants and was used the same number of times as the first and second sequence (i.e., 2).

#### Procedure

Participants were seated approximately 60 cm from the screen. Participants were required to name the items (pictures or words) preceded by the corresponding definitive determiner. There was no familiarization phase. The experimental session consisted of a total of two sequences of 148 trials; there was a short pause between sequences. Participants were not corrected by the experimenter throughout the experimental session. An experimental trial consisted of the following events. A fixation cross was shown in the center of the screen. In order to prevent participants from falling into a rhythm about when they were producing responses, the duration of the fixation cross was (randomly) varied among four durations: 300, 400, 500, and 600 ms. The fixation cross was followed by a blank screen for 500 ms. Following the blank screen the target picture or word was presented for 2000 ms or until the participant’s response. Response latencies were measured from the onset of the picture. The next trial began 1500 ms after the onset of participants’ response. Stimulus presentation, response times and response recording were controlled by the program DMDX (Forster and Forster, 2003). Naming latencies and accuracy were determined using the CheckVocal software (Protopapas, 2007).

#### Analysis

Analyses were performed on critical items only and separately for pictures and words. Production of clearly erroneous picture names and verbal dysfluencies were excluded from the analysis of response times and considered as errors. A total of 3.7% of the data points were excluded following these criteria. In addition, voice-key failures (0.1%) were removed from the analysis. Mean naming latencies and error rates by condition are reported in **Table 2**. Naming latencies were log-transformed using the natural logarithm to reduce skewness and approximate a normal distribution. We performed two analyses on the naming latencies. We first tested for the presence of a cumulative semantic cost in determiner + picture naming and determiner + word naming separately. This analysis was performed on non-deviant trials only. That is, for determiner + picture naming trials, the first, second and third ordinal positions for the conditions PPP-P and PPP-W, and the fourth ordinal position of the condition PPP-P were included in the analysis. For determiner + word naming trials, the first, second and third ordinal position of the conditions WWW-W and WWW-P and the fourth ordinal position of the condition WWW-W were included in the analysis. We expected to replicate the cumulative semantic cost with target pictures (e.g., Howard et al., 2006) but not with word targets (Navarrete et al., 2010; Belke, 2013).

**Table 2:** Mean naming latencies (RT in ms), standard deviations (SD in ms) and percentage of error rates (E) by Ordinal Position Within-Category and Condition.

	Conditions											
	PPP-P			PPP-W			WWW-W			WWW-P		
Position	*RT*	*SD*	*E*	*RT*	*SD*	*E*	*RT*	*SD*	*E*	*RT*	*SD*	*E*
(1)	724	183	5.8	707	160	5.8	521	102	0.3	517	99	0.5
(2)	751	211	5.6	754	217	5.6	517	96	0.8	522	100	1.3
(3)	762	221	9	769	207	9.6	517	98	0.8	517	112	1
(4)	787	224	8.7	523	94	0.3	510	99	0.8	747	193	8.1
*Mean*	*755*		*7.3*	*685*		*5.3*	*516*		*0.6*	*516*		*2.7*

In a second analysis, we explored the transfer of semantic interference from determiner + picture naming to determiner + word naming trials and from determiner + word naming to determiner + picture naming trials. To this end, for pictures, we compared naming latencies in the fourth ordinal position in the PPP-P condition (i.e., non-deviant trials) to naming latencies in the fourth ordinal position in the WWW-P condition (i.e., deviant trials). The same was done for words, that is, we compared naming latencies in the fourth ordinal position in the WWW-W condition (i.e., non-deviant trials) to naming latencies in the fourth ordinal position in the PPP-W condition (i.e., deviant trials). We expected to replicate our previous study. That is, for picture trials, faster naming latencies for deviant picture trials than for non-deviant picture trials; for word trials, slower naming latencies for deviant word trials than for non-deviant word trials.

Analyses were performed employing linear mixed-effects models (LMM) with crossed random effects for participant and items. We used the lme4 package (Bates et al., 2011) with the R program (R Development Core Team, 2011). For the first analysis, the following LMM models were tested and compared separately for picture naming trials and word naming trials. The null models (see 0_Cumulative models) contained intercepts only and no predictors. First we added the predictor Condition (see 1_Cumulative models). We then included the predictor Lag (see 2_Cumulative models). Finally, we added the critical predictor Order within-category in order to explore the cumulative semantic cost (see 3_Cumulative models), separately for pictures and word naming trials. For the second type of analysis, that is, for trials from the fourth within-category ordinal positions, the same logic was applied. The null model (see 0_Transfer models) contained intercepts only and no predictors. First the predictor Lag was added (see 1_Transfer models) and afterward, the critical Condition (see 2_Transfer models) was included in order to explore differences between deviant and non-deviant naming trials, again separately for picture and word trials. In each of these models the same random effects were set: participants and items. The comparison between models was performed on the likelihood ratio test and took into consideration the Bayesian information criterion (BIC; Schwarz, 1978). We calculated the BIC difference (ΔBIC) between the null model and the other models. We then used the Bayes factor (BF) approximation formula [exp(ΔBIC/2); Raftery, 1995] to compare the relative evidence of the different models. In general, the higher the BF and the Δbic, the more evidence there is for the model compared to the null model.

### Results

#### Picture Naming Trials

Analysis were performed on 2981 data points. As shown in **Table 3**, model P1_Cumulative does not improve the fit in relation to the null model. In contrast, the inclusion of the predictor Lag (P2_Cumulative) improves the model fit. We therefore kept Lag as a critical predictor and explored the influence of Order within-category. The model with the predictor Order within-category (P3_Cumulative) shows a better fit in relation to the model with Lag as the unique predictor. This last result replicated the cumulative semantic cost in determiner + picture naming (Navarrete et al., 2010; Belke, 2013), naming latencies increase with each additional within category item that is named (see **Table 2**). In relation to transfer effects, as can be seen in **Table 2**, naming latencies were faster for deviant trials (747 ms, fourth ordinal position in the WWW-P condition) than in non-deviant trials (787, fourth ordinal position in the PPP-P condition). Models testing this effect are reported at the bottom of the **Table 3**. The model with the critical predictor condition, P2_Transfer, shows a better fit in relation to the null model and the model with the predictor Lag (P1_Transfer).

**Table 3:** The fit indices on the analysis with picture trials.

	Fixed effects	Model Df	Chisq (df)	*p*	BIC	Δbic	Approx. BF
P0_Cumulative		4			-1184		
P1_Cumulative	Condition	5	4 (1)	=0.03	-1181	-3	0.2
P2_Cumulative	Lag	5	47 (1)	<0.001	-1224	40	>10000
P3_Cumulative	Lag + order within-category	6	71(2)	<0.001	-1241	57	>10000
P0_Transfer		4			-175		
P1_Transfer	Lag	5	0.5(1)	=0.49	-169	-6	0.04
P2_Transfer	Condition	5	7	<0.01	-176	1	1.7

*Df, degree of freedom; Chisq (df), chi-squared and degree of freedom; p, probability value; BIC, Bayesian Information Criterion; Δbic, differences between the Null Models (i.e., P0_) and other models; Approximately BF, Bayes Factor’s (BF) approximation, exp(Δbic/2).*

#### Word Naming Trials

Analysis were performed on 3177 data points. As shown in **Table 4**, the null model (W0_Cumulative) with only participants and items as random predictors shows a better fit in relation to the models that contained Condition, Lag and Order within-category as predictors (W1_Cumulative, W2_Cumulative and W3_Cumulative models). In other words, this result suggests that there is no cumulative semantic cost in determiner + word naming task, replicating previous findings (Navarrete et al., 2010; Belke, 2013). In relation to transfer effects, as can be seen in **Table 2**, naming latencies were slower for deviant trials (523 ms, fourth ordinal position in the PPP-W condition) than for non-deviant trials (510 ms, fourth ordinal position in the WWW-W condition). The model with the critical predictor of condition, W2_Transfer, shows a better fit in relation to the null model, indicating that there was a transfer of semantic interference from determiner + picture naming to determiner + word naming trials (see bottom at **Table 4**).

**Table 4:** The fit indices on the analysis for word trials.

	Fixed effects	Model Df	Chisq (df)	*p*	BIC	Δbic	Approx. BF
W0_Cumulative		4			-3419		
W1_Cumulative	Condition	5	0.54 (1)	=0.46	-3412	-7	0.03
W2_Cumulative	Lag	5	17(1)	= 68	-3411	-8	0.01
W3_Cumulative	Order within-category	5	5.6 (1)	<0.02	-3416	-3	0.22
W0_Transfer		4			-910		
W2_Transfer	Lag	5	0.2(1)	=0.61	-904	-6	0.04
W3_Transfer	Condition	5	10 (1)	<002	-914	4	7

*For the fit statistics, see the note to **Table 3**.*

### Discussion

**FIGURE 1:**
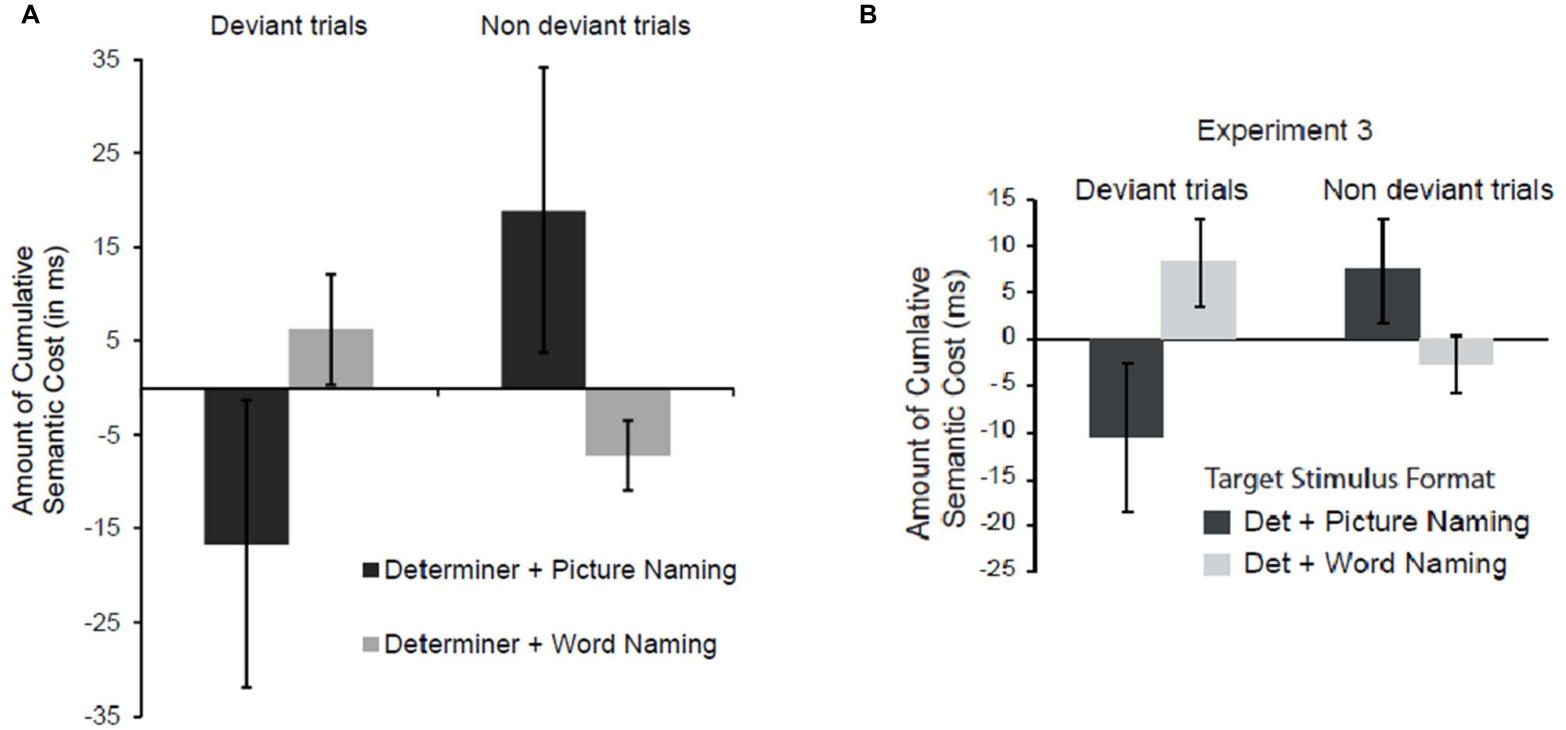
**(A)** Mean differences in naming latencies representing the amount of transfer of interference observed for deviant and non-deviant trials (i.e., in the fourth ordinal position), for pictures and word targets. The difference was computed as the difference score between trials at the fourth ordinal position and trials at the third ordinal position within the same format (i.e., picture or word). Specifically, for deviant picture targets we calculated the difference scores between deviant trials (i.e., fourth ordinal position in the WWW-P condition) and trials in ‘*n-1*’ ordinal position (i.e., third ordinal position in the PPP-P condition); while for non-deviant picture targets we calculated the difference scores between non-deviant trials (i.e., fourth ordinal position in the PPP-P condition) and trials in ‘*n-1*’ ordinal position (i.e., third ordinal position in the PPP-P condition). The same was done for word trials. For deviant word targets we calculated the difference scores between deviant trials (i.e., fourth ordinal position in the PPP-W condition) and trials in ‘*n-1*’ ordinal position (i.e., third ordinal position in the WWW-W condition); while for non-deviant word targets we calculated the difference scores between non-deviant trials (i.e., fourth ordinal position in the WWW-W condition) and trials in ‘*n-1*’ ordinal position (i.e., third ordinal position in the WWW-W condition). Difference scores were calculated on a subject-by-subject basis (see Navarrete et al., 2010 for details). A positive value reflects a transfer of interference for consecutive ordinal positions within-category. **(B)** Transfer effects reported by (Navarrete et al., 2010; Experiment 3).

This pattern of results replicates the main findings of Experiment 3 in Navarrete et al. (2010). In our previous Experiment, we showed that semantic interference is transferred from determiner + picture naming to determiner + word naming, but not from determiner + word naming to determiner + picture naming. We explained this pattern by suggesting that determiner + word naming does not involve semantically driven lexical access, but does require lexical access. This would explain why determiner + word naming trials are affected when previous within-category items were determiner + picture naming trials. At the same time, determiner + word naming does not involve incremental weakening of the semantic-to-lexical connections for semantic coordinate items, explaining the lack of transfer of the semantic interference in determiner + picture naming trials when the preceding within-category trials are determiner + word naming trials. This pattern was replicated in the present experiment. As can be seen in **Figure 1**, the conditions showing a transfer of interference were the non-deviant picture condition (determiner + picture following determiner + picture naming trials) and the deviant word condition (determiner + word following determiner + picture naming trials). No transfer of interference was reported in the deviant picture condition (determiner + picture following determiner + word naming trials) or the non-deviant word condition (determiner + word following determiner + word naming trials; for a similar pattern see Figure 4 in Navarrete et al., 2010).

## General Discussion

An important open issue in the field of lexical access concerns the origin(s) of semantic context effects in language production tasks. As reviewed in the Introduction, studies exploring semantic effects have revealed a consistent pattern with picture stimuli, but the evidence for words is sparse and inconsistent. In the experiment reported here we have explored whether pictures and words elicit similar long lasting *inter-trial* semantic interference effects in language production. Participants named pictures and words along with their gender marked definite determiner. The presentation format (picture or word) of the target stimuli varied within the semantic categories. For some categories, all the within-category items were presented in picture format, while for other categories, all the within-category items were presented in word format. A cumulative semantic cost with picture stimuli was observed, such that naming times increased with each additional within-category item that was named, replicating previous findings (e.g., Brown, 1981; Howard et al., 2006). In contrast, no cumulative semantic cost was reported with word stimuli, that is, when all of the within-category items were word targets, replicating previous findings (Navarrete et al., 2010; Belke, 2013).

Furthermore, two other conditions were tested. For some categories, the last within-category item was presented in a different format (picture or word) from the other previously presented within-category items (picture or word). These two conditions allow us to explore the transfer of semantic interference from picture to word naming trials and vice-versa, from word to picture naming trials. As stated above, whether or not there is semantic interference transfer between words and pictures is a critical issue for current models of lexical retrieval. The results demonstrated a transfer from picture to word naming trials, but not in the other direction, from word to picture naming trials. That is, while determiner + picture naming trials induce a cumulative semantic cost for subsequent within-category named determiner + word naming trials, determiner + word naming trials do not induce a semantic cost for subsequent within-category determiner + picture naming trials, replicating previous findings (Navarrete et al., 2010; see Experiment 3). This pattern suggests that lexical retrieval can be accomplished differently, depending on the format of the target stimuli. When the target is a picture, lexical retrieval is a semantically mediated process and semantic-to-lexical connections will be adjusted: connections from semantics to words are strengthened for the target word and weakened for semantic coordinates of the target word (Oppenheim et al., 2010). In contrast, when the target is a word, connections between semantics to words will (probably) be strengthened for the target word but not weakened for semantic coordinates of the target word. As a consequence, there is no semantic interference (i.e., cumulative semantic cost) for the subsequent within-category item, regardless of its format (picture or word). In sum, the transfer of semantic interference from picture to word trials suggests that determiner + word naming involves lexical access, while the lack of transfer of semantic interference from word to picture (and word) trials suggest that determiner + word naming does not weaken the semantic-to-lexical connections of semantic coordinates of the target word (Navarrete et al., 2010)^3^.

Interference induced by having previously retrieved semantically related information is a broader phenomenon. For instance, retrieval-induced forgetting is a phenomenon in which the recall of a previously studied word is hampered if, between the learning and the recall phase, participants are required to ‘actively’ retrieve other exemplars from the same semantic category (e.g., Anderson, 2003). That is, retrieval of unpracticed items from practiced categories is worse than retrieval of unpracticed items from unpracticed categories. Critically, a factor that determines retrieval-induced forgetting is the format in which the practice exemplars are presented. The phenomenon appears, for instance, when participants are presented with ‘part of the word’, as in the case of category-stem cues such as ‘FRUIT-or___’, and have to ‘actively’ retrieve the word (orange). However, when participants do not ‘actively’ retrieve the word but simple read it, by means of, for instance, category-stem cues such as ‘FRUIT-orange’, no retrieval-induced forgetting is observed (Anderson et al., 2000). The origin of long lasting *inter-trial* semantic interference is generally considered within the somewhat narrow scope of language production processes. However, the parallels with other phenomena, such as retrieval-induced forgetting, suggest that it may be promising to take a broader view. In this line, the results reported here would be congruent with the notion that only when lexical retrieval entails an ‘active’ process, as in the case of picture naming trials, there are incremental learning adjustments to the semantic-to-lexical connections and long lasting semantic interference (i.e., cumulative semantic cost) is propagated to subsequent within-category items. Such adjustments are not present when lexical retrieval does not require ‘active’ lexical retrieval, as in the case of word naming trials (Navarrete et al., 2010; see also Oppenheim et al., 2010).

In comparison to language comprehension research, it is more difficult to control the input stimulus in speech production experiments. While it is relatively easy to control relevant variables of the input words in comprehension tasks, it is harder to elicit the expected responses in production tasks. This could be a reason why written words have been extensively used as target stimuli in speech production research. Another reason could be that the most influential model of lexical retrieval in speech production, the model developed by Levelt et al. (1999), has localized semantic interference effects at the lexical level. As written words have a direct link to the lexical system (i.e., lemma and/or lexeme), they modulate the processes occurring at the lexical level of processing (for discussion see Roelofs et al., 1996). This theoretical approach has resulted in an increased use of written words as experimental stimuli, in both the *inter-trial* and *intra-trial* semantic context manipulations. However, our findings indicate that the format of the target stimuli is a critical factor that has to be taken into consideration.

## Conclusion

Our results suggest that long lasting *inter-trial* semantic interference is caused by adjustments to the semantic-to-lexical connections that occur in picture naming, but which do not occur in word naming. Consistent with prior arguments (Vitkovitch and Humphreys, 1991; Navarrete et al., 2010; Oppenheim et al., 2010; see also Kleinman et al., 2015) we suggest that long lasting *inter-trial* semantic interference in language production arises as a consequence of incremental adjustments to semantic-to-lexical connections, and that such adjustments to not obligatorily occur for word reading.

## Conflict of Interest Statement

The authors declare that the research was conducted in the absence of any commercial or financial relationships that could be construed as a potential conflict of interest.

## Footnotes

1 Howard et al. (2006) do not make specific predictions regarding transfer effects between word and picture targets. It is however worth mentioning that Howard et al. (2006) account would predict a transfer of semantic interference from picture to word naming. This would be so because if prior picture naming events modify the accessibility of a given lexical unit, this should generate interference when the subsequent within-category trial requires lexical access, as it is the case in a determiner + word naming task.

2 P stands for ‘picture format’ and W for ‘word format’. The names of the conditions (e.g., PPP-P) do not refer to the presentation format of four consecutive trials within the sequence, but the presentation format of the four within-category items (e.g., in PPP-P all within-category items were pictures).

3 Although grammatical gender in Italian nouns is basically a lexical property and is not related with noun meaning and phonological forms, there are some regularities between the phonological noun endings and grammatical gender. For example, Italian nouns ending in –o are predominantly masculine while nouns ending in –a are predominantly feminine. Based on this, it could be argued that on some word trials, participants might be accessing the appropriate determiner based on word ending information only. If this were the case, this could explain the lack of transfer of semantic interference with word targets, because accessing determiners from word ending information bypasses lexical retrieval. Critically, this would be independent of the experimental condition. That is, it would be independent of whether a target word is located at the fourth within-category ordinal position in condition WWW-W or at the fourth within-category ordinal position in condition PPP-W. Therefore, no semantic interference effects would be expected in either condition. That is not what the results showed. That is, the transfer of semantic interference from pictures to words in the PPP-W condition ensures that participants were accessing the corresponding lexical representation in the word target trials.
