# Supplementary material for: What can Written-Words Tell us About Lexical Retrieval in Speech Production?
Source: Front Psychol. 2016 Jan 6;6:1982. doi: 10.3389/fpsyg.2015.01982 (PMC4701968; doi:10.3389/fpsyg.2015.01982)
Supplement: Supplementary file 1 [file Data_Sheet_1.DOCX]

**Appendix**

Experimenal materials organized by semantic category.

Tools: drill, hammer, saw, screwdriver.

Transport: truck, motorcycle, camper, train.

Tableware: ladle, fork, knife, spoon.

Furniture: bed, chair, desk, bench.

Farm animals: cow, sheep, horse, pig.

Vegetables: eggplant, carrot, zucchini, onion.

Body parts: nose, mouth, finger, hand.

Clothes: pants, jacket, shirt, sweater.

White goods: dishwasher, fridge, microwave, washing machine.

Buildings: castle, church, home, windmill.

Fruits: apple, banana, lemon, cherry.

Musical instruments: drum, guitar, piano, trumpet.

Computer equipment: computer, joystick, keyboard, mouse.

Birds: hen, parrot, swan, owl.

Bugs: mosquito, ladybug, butterfly, fly.

Landscape features: waterfall, desert, sea, mountain.

Weapons: cannon, rifle, miter, gun.

Reptiles and amphibians: crocodile, frog, lizard, snake.

House parts: stairs, fireplace, roof, window.

Celestial phenomena: cloud, sun, moon, lightning.
